# Towards Harmonious Coexistence in the Unlicensed Spectrum: Rational Cooperation of Operators

**DOI:** 10.3390/s17102432

**Published:** 2017-10-24

**Authors:** Sunghwan Bae, Hongseok Kim

**Affiliations:** Department of Electronic Engineering, Sogang University, Seoul 04107, Korea; usher731@sogang.ac.kr

**Keywords:** 5G New Radio (NR), long-term evolution (LTE), LTE-license assisted access (LTE-LAA), LTE in unlicensed spectrum (LTE-U), listen-before-talk (LBT), clear channel assessment (CCA), operator’s dilemma, game theory

## Abstract

5G New Radio (NR) operating in the unlicensed spectrum is accelerating the Fourth Industrial Revolution by supporting Internet of Things (IoT) networks or Industrial IoT deployments. Specifically, LTE-Advanced (LTE-A) is looking to achieve spectrum integration through coexistence with multi-radio access technology (RAT) systems in the same unlicensed bands with both licensed-assisted and stand-alone access. The listen-before-talk (LBT) mechanism is mainly considered to enable an LTE operator to protect other incumbent unlicensed systems. In this article, we investigate the behaviors of multiple LTE operators along with the deployment of WiFi networks in the unlicensed spectrum from both short- and long-term points of view. In countries without mandatory LBT requirements, we show that an LTE operator is susceptible to collusion with another LTE operator, thus exploiting scarce spectrum resources by deceiving other wireless networks into thinking that channels are always busy; hence, mandatory usage of LTE with LBT is highly recommended at national level to achieve harmonious coexistence in the unlicensed spectrum. We discuss several possible coexistence scenarios to resolve the operator’s dilemmaas well as to improve unlicensed spectrum efficiency among multi-RAT systems, which is viable in the near future.

## 1. Introduction

### 1.1. The Next Generation Wireless Technologies and Spectrum Scarcity

Ever increasing traffic demand and ultra-low latency requirements have driven technological advancements in wireless communications, namely 5G New Radio (NR) [1]. The previous 4G network deployments with Long-Term Evolution-Advanced (LTE-A) have successfully offered not only great quality of service (QoS), high spectral efficiency, and long-range coverage [2,3], but also a myriad of new business opportunities in different industries such as smart grid, healthcare, and Internet of Things (IoT) [4,5]. Along with the Fourth Industrial Revolution, to support ultra-high capacity and ultra-low latency wireless systems, key enabling technologies such as ultra-dense small cell networks, massive multiple-input multiple-output (MIMO), and full-duplex communications are just around the corner [6]. In addition, new spectrum usage paradigms have been considered to make more spectrum resources available to 5G-related services because spectrum scarcity is expected to be one of the fundamental bottlenecks in 5G NR [7].

### 1.2. The Expansion of Spectrum for Wireless Systems

To address the issue of spectrum scarcity, cognitive radio (CR) can utilize the unused spectrum bands of a primary user (PU) in an opportunistic manner as long as secondary users (SUs) guarantee no harmful interference [7]. Recent migration from analog to digital television broadcasting has expedited the opportunistic sharing of underutilized TV spectrum bands called TV white space (TVWS) [7]. For example, IEEE 802.22 and IEEE 802.11af standards opportunistically utilize TVWS, and the US Federal Communications Commission (FCC) and the UK Office of Communication (OFCOM) have updated rules for TVWS and other unlicensed bands [7]. As a cellular application of CR technology, *LTE-CR* was proposed to extend the LTE time-division duplex (TDD), also known as TD-LTE to support TVWS [8]. In [9], the authors applied cooperative sensing mechanisms of CR to the LTE frequency-division duplex (FDD) with an almost blank subframe (ABS) to achieve the maximum utilization of TVWS. In [10], optimal cooperative sensing under the heterogeneous received signal to noise ratio was investigated. Moreover, standardization bodies recently developed ideas on LTE in the unlicensed spectrum such that LTE-A is looking to achieve spectrum integration through coexistence with multi-radio access technology (RAT) systems in the same unlicensed bands [11].

### 1.3. LTE-U and LTE-LAA

The LTE-unlicensed (LTE-U) initiative by Qualcomm has led to a standard for operating LTE-A over the unlicensed spectrum. The LTE-U Forum focuses on supplemental downlink (SDL) mode of LTE in the unlicensed spectrum paired with a licensed LTE spectrum by using carrier aggregation (CA) [12]. Since stand-alone access to unlicensed spectrum may be unstable due to the unknown interference, 3GPP has worked on a single global solution framework for LTE licensed-assisted access (LTE-LAA) to unlicensed spectrum, targeting fair coexistence with existing WiFi networks in the 5-GHz spectrum [13]. To determine whether a channel of the unlicensed spectrum is occupied or not, it is necessary for LTE operators to use listen-before-talk (LBT), which is one of the clear channel assessment (CCA) mechanisms for carrier sensing before transmission [13]. Although European and Japanese regulations mandate LBT features in the unlicensed bands, other countries such as the United States, China, and South Korea are still considering it [14].

### 1.4. Related Work and Motivation of LTE Operators’ Behavior in the Unlicensed Spectrum

Different LBT deployments were used in [14] to analyze the average user throughput of LTE small cell networks and to verify how WiFi systems are affected by LTE in the unlicensed spectrum. The authors of [15] evaluated throughput performance for both LTE with LBT and WiFi systems under indoor and outdoor scenarios based on stochastic geometry. In [16], extensive simulations were performed to investigate the effectiveness of adjusting transmission time for LTE-LAA with coexisting WiFi networks based on the indirect estimation of the data rate and traffic load of WiFi networks. The work of [17] has evaluated various LBT schemes such as 3GPP category 4 LBT and extended clear channel assessment (ECCA) to quantify the performance tradeoff of WiFi networks. According to [18], indoor-based interference simulations have addressed channel-sharing issues between LTE-LAA and a WiFi network, and different bandwidths of LTE-LAA (1.4/3/5/10 MHz) have shown different throughput degradation of WiFi in experiments. The authors of [19] verified the optimal transmission time of LTE-LAA by considering a MAC protocol design to protect WiFi in the unlicensed spectrum. In [20], the performance of two coexisting WiFi networks were investigated first, and then one of the WiFi networks was replaced with an LTE operator to quantify the existing WiFi network’s user-perceived throughput (UPT) which is the amount of traffic over the actual transmission time excluding the idle time. In [21], the authors extensively presented a classification of different techniques that can be applied on co-located LTE and Wi-Fi networks. The work of [22] also investigated the coexistence of LTE and WiFi where the LTE base station uses WiFi-like carrier sense, back-off, and Quality of Service facility techniques. In [23], the impact of coexistence on WiFi performance and LTE performance was analyzed. According to [13], studies on the operation of LTE in the unlicensed spectrum concluded that LTE-LAA and WiFi systems can harmoniously coexist because LTE-LAA using LBT causes less adjacent channel interference to a WiFi network compared to another WiFi network.

Despite research on coexistence between LTE and WiFi systems, scenarios involving multiple LTE operators in the unlicensed spectrum have not yet been well studied from theoretical and practical perspectives. Unlicensed spectrum debates in Korea highlighted that an opportunity for the 5 GHz band is likely to induce a new paradigm of LTE operators’ policies on the competitive environment [24]. To this end, this article first verifies the interaction between multi-RAT systems the in unlicensed spectrum and then determines the best strategy to maximize their utility. Specifically, we focus on the behavior of two operators with user-deployed WiFi networks based on the framework of game theory. Game theory is a useful tool for modeling independent decision makers whose actions affect others’ decisions [25]. Many issues related to wireless communications have been extensively examined by game theory to establish intuition about the strategic operation of systems from both short- and long-term points of view [26]. To this end, we introduce the operator’s dilemma and discuss how to improve the efficiency of spectrum utilization to achieve harmonious coexistence between multi-RAT systems in the unlicensed spectrum.

The rest of our paper is organized as follows. In Section 2, basic operation mechanisms for LTE operators and WiFi networks are introduced. The LTE operators’ dilemma in the unlicensed spectrum is addressed with the game theory framework, and performance analysis is represented in Section 3. Finally, we draw our conclusions with discussion and future topics in Section 4.

## 2. Multi-RAT Systems in the Unlicensed Spectrum

### 2.1. LTE Operators and WiFi Networks in the Unlicensed Spectrum

**Figure 1:**
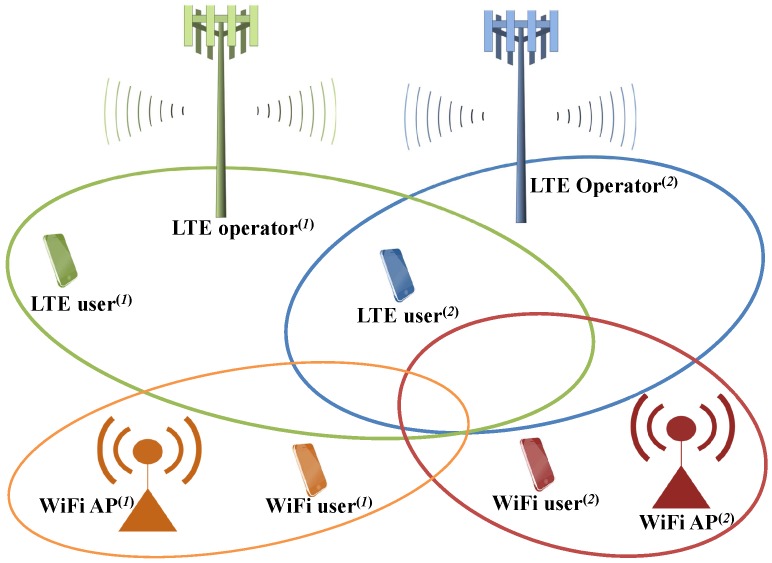
A scenario of multi-radio access technology (RAT) systems operating in the unlicensed spectrum: there are two Long-Term Evolution (LTE) operators and two user-deployed WiFi networks.

Suppose that there are four wireless networks utilizing unlicensed spectrum, e.g., two different LTE operators and two user-deployed WiFi networks as shown in Figure 1. To verify the cooperative or competitive behavior of networks, we assume that all networks use the same channel bandwidth of 20 MHz in 5 GHz unlicensed spectrum. For those countries where LBT schemes on the unlicensed spectrum are mandated by telecommunications regulatory bodies, reciprocal LTE operators always perform their own LBT schemes to protect other incumbent networks to achieve harmonious coexistence, e.g., LTE with LBT.

For this reason, existing literature has assumed mutual benefits in the unlicensed spectrum and focused on the performance evaluation of either LTE operators or WiFi networks under various LBT schemes [14,15,16,17,18,19,20]. Nevertheless, for those countries where the LBT mechanism is not mandatory, would LTE operators still have the incentive to perform their own LBT schemes in the unlicensed spectrum? Here, we would rather focus on the potential behaviors of LTE operators based on whether an LBT scheme is necessarily mandated or not. When the LBT scheme is not mandatory, LTE operators have two alternatives to utilize the unlicensed spectrum; LTE with LBT is one option. The other option is LTE-itself based on a centralized MAC protocol. An LTE operator that takes LTE-itself can immediately schedule resource blocks (RBs) to its users in every subframe to maximize its own target metric. Obviously, there is a possibility that the selfish LTE operator exploits the unlicensed spectrum without considering other LTE and WiFi networks. For example, the selfish LTE operator may decide not to use the LBT scheme so that it can save energy and time for spectrum sensing. WiFi networks always take reciprocal action because they originally operate based on the distributed coordination function (DCF) which is a contention-based CCA adopting carrier sense multiple access with collision avoidance (CSMA/CA) [26]. To this end, we introduce an unlicensed spectrum game with two LTE operators using two strategies to investigate their behaviors in the unlicensed spectrum.

### 2.2. Payoffs of LTE and WiFi Networks

Although WiFi networks cannot be considered as a player in this game because they have only one strategy, we consider payoffs of WiFi networks depending on the behaviors of LTE operators. Before formulating a game, we first generalize payoffs of both LTE and WiFi operators as follows.

$U_{s}$ is the payoff when a single network exploits one channel of the unlicensed spectrum and has a successful transmission in a given time period. If $U_{s}$ is considered as the channel capacity of a certain network, it depends on radio access technology (RAT), inherent RF performance, the number of users, scheduling methods, and geographical conditions. We also denote $U_{s}^{LTE}$ and $U_{s}^{WiFi}$ as payoffs of LTE and WiFi networks, respectively.

$U_{sh}$ is the payoff that all networks harmoniously share the co-channel of the unlicensed spectrum by using CCA. The exact values of $U_{sh}$ for multi-RATs are different because of CCA schemes and scheduling methods configured by a certain network. Regardless of networks’ characteristics, $U_{s}>U_{sh}$ generally holds because $U_{sh}$ results from multi-RATs coexisting in the co-channel of the unlicensed spectrum. In the sharing scenario, $U_{sh}^{LTE}$ and $U_{sh}^{WiFi}$ are payoffs of LTE and WiFi networks, respectively and usually $U_{sh}^{LTE}>U_{sh}^{WiFi}$ holds based on experiments [14]. The reason is that as soon as the LBT procedure is used by an operator, LTE users can access the unlicensed spectrum by centralized LTE scheduling, which has significantly higher spectral efficiency than the DCF mechanism [14].

$U_{f}$ is the payoff that two LTE operators fail to transmit data by causing severe unknown interference to each other when they use the co-channel of the unlicensed spectrum at the same time. Although this undesirable case may provide a weak communication link between users and an operator, no operators want to encounter low throughput with unreliable transmission. In addition, there is a possibility that imperfect sensing of new arrival networks induces the hidden node problem such that multi-RATs networks interfere each other spatially and temporally. To focus on the behavior of LTE operators, herein, we assume that all systems are capable of perfect sensing under the CCA procedure.

$U_{w}$ is the payoff when LTE and WiFi networks wait for the next transmission opportunity. In this case, both networks cannot utilize the unlicensed spectrum based on its CCA result even if they have full of buffer data to transmit. Here, note that the term CCA includes CSMA/CA as well as many LBT schemes such as carrier-sensing adaptive transmission (CSAT), LBT with duty cycle, and LBT with random back-off [14]. Moreover, the value of $U_{w}$ can be zero in terms of channel capacity when the network is idle in the unlicensed spectrum. However, for high-quality real-time traffic such as voice and video streaming applications, mobile subscribers are extremely delay-sensitive such that the potential revenue loss due to frequent and/or long delay is considered as an opportunity cost to LTE operators, and thus $U_{w}$ can be negative.

## 3. Operator’s Dilemma in the Unlicensed Spectrum

**Figure 2:**
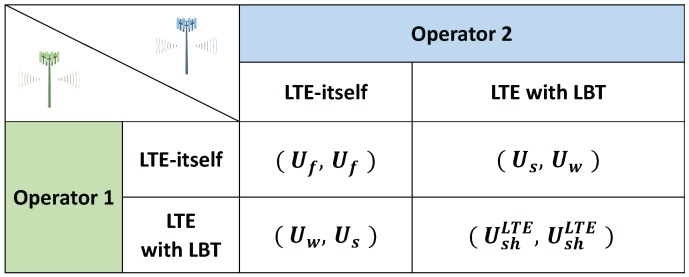
The payoff matrix of wireless systems in the unlicensed spectrum: two LTE operators coexist.

**Figure 3:**
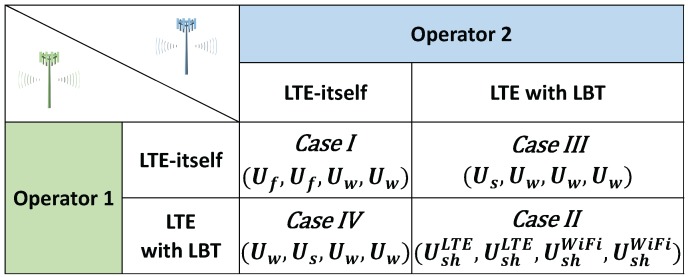
The payoff matrix of wireless systems in the unlicensed spectrum: Two LTE operators and two WiFi networks coexist.

Generally, $U_{s}>U_{sh}>U_{f}>U_{w}$ holds and we focus on the behaviors of two LTE operators. The payoff matrix of Figure 2 shows how the game continues when only LTE operators simultaneously play. However, since WiFi networks are inevitable in the unlicensed spectrum, LTE operators should consider WiFi networks in multi-RAT systems. Even though a WiFi network cannot be a player, we include payoffs of two WiFi networks as dummy variables in Figure 3. In each case, an element of a payoff vector represents LTE operator 1’s payoff, LTE operator 2’s payoff, WiFi network 1’s payoff, and WiFi network 2’s payoff, respectively.

### 3.1. Preliminary of the Unlicensed Spectrum Game

The unlicensed spectrum game works as follows. One operator chooses either LTE-itself or LTE with LBT, as does the other operator simultaneously. These choices result in one of the four possible outcomes shown in Figure 3 together with two WiFi networks. For WiFi networks, their payoffs are accordingly given by the action of two LTE operators.

*Case I* If both LTE operators egoistically use the co-channel of the unlicensed spectrum as LTE-itself, both operators get $U_{f}$ corresponding to severe unknown interference. As a result, two LTE operators are punished due to mutual defection, e.g., not using LBT; also, two WiFi networks have no opportunity to communicate. *Case II* If both LTE operators use proper LBT schemes, all networks can share the unlicensed spectrum harmoniously by getting a meaningful portion of time slots for reliable communications. Thus, $U_{sh}^{LTE}$ and $U_{sh}^{WiFi}$ are the rewards for mutual cooperation. *Case III* If LTE operator 2 plays as LTE with LBT and the other sticks to LTE-itself, then operator 1 takes $U_{s}$, but operator 2 as well as two WiFi networks have $U_{w}$. Finally, *Case IV* is the opposite of *Case III*.

### 3.2. One-Shot Game in the Unlicensed Spectrum

Our goal is to find the best strategies of two LTE operators in the unlicensed spectrum one-shot game. Suppose that LTE operator 1 thinks that LTE operator 2 will use the LBT scheme. At this time, we notice that LTE operator 1 will get $U_{s}$ of the temptation payoff by using LTE-itself (*Case III*). On the other hand, operator 1 can also use the LBT scheme, achieving $U_{sh}^{LTE}$ of the reward for mutual cooperation (*Case II*). It results in better outcomes for WiFi networks too. However, since we assume that LTE operators are rational decision-makers, LTE operator 1 will choose LTE-itself because $U_{s}>U_{sh}^{LTE}$ always holds. Now suppose that LTE operator 1 guesses that LTE operator 2 will be LTE-itself. LTE operator 1 has a choice between LTE-itself and LTE with LBT; consequently, it will get either $U_{f}$ or $U_{w}$. Thus, when LTE operator 1 is sure that LTE operator 2 will choose LTE-itself, LTE-itself will be the best strategy for LTE operator 1 because $U_{f}>U_{w}$ holds (*Case I*).

Consequently, no matter what the other operator does, choosing LTE-itself is obviously better for LTE operator 1, and vice versa for LTE operator 2. Both LTE operators are expected to take LTE-itself and will eventually get $U_{f}$ which is worse than $U_{sh}^{LTE}$ from the point of view of harmonious coexistence. To summarize the one-shot game in the unlicensed spectrum, we conclude that operators’ rationality leads to a worse result for themselves as well as WiFi networks—called the operator’s dilemma. Although exact values of payoffs are not explicitly described here, the operator’s dilemma follows a similar logic to the prisoner’s dilemma but with additional WiFi networks. The prisoner’s dilemma is a well-known example in game theory that shows why two completely rational individuals might not cooperate, even if it is in their best interests to do so [25]. Next, we focus on how this dilemma can be resolved in several repeated games.

### 3.3. Finite Repeated Game in the Unlicensed Spectrum

Now we investigate whether two LTE operators have any incentive to take LTE with the LBT strategy, especially when the game is played a known finite number of times. We assume that two LTE operators are aware of the end of the game and monitor the other operator’s action perfectly.

Note that there is significant difference between the one-shot game and repeated game. The repeated game can induce punishment, retaliation, collusion, and cooperation that are not possible choices for operators in the one-shot game. First, we focus on two LTE operators repeating the previous one-shot game a finite number of times *T*. To solve a finite horizon game, backward induction is useful to verify the behavior of operators. Intuitively, if two LTE operators encounter the last stage of the game, they choose the dominant LTE-itself strategy because there is no future on which to have an influence themselves. This means that two LTE operators still have no incentive to harmoniously coexist with a known finite number of times. Based on the knowledge of backward induction, by going back through the stages of the game, two LTE operators select the dominant LTE-itself strategy in *every* sequence of the game. However, this conclusion would not apply if operators can play an infinite number of times. In reality, since cellular networks constantly operate to provide communications services, it is not possible for everlasting operators to know the last moment of the game.

### 3.4. Infinite Repeated Game in the Unlicensed Spectrum

First, we define an operator’s utility for playing an infinitely repeated game as follows.

**Definition** **1.** *For operator i, given an infinite stage of payoffs $U_{i1},U_{i2},U_{i3},\cdots ,U_{ik},\cdots$, and discount factor $\delta_{i}$ with $0<\delta_{i}<1,$ i’s future discounted reward is*

(1) $$\sum_{k=1}^{\infty }\delta_{i}^{k-1}U_{ik}.$$

Assuming a common discount factor for all operators, e.g., $\delta_{i}=\delta$ for all *i*, the discount factor δ has two equivalent interpretations. First, δ means that the operator *i* tends to care about its payoffs in the near term more than that in the long term. The other interpretation of δ is such that the operator cares about the future just as much as the present, but with probability 1−δ, the game will end in any given round, e.g., there is always some chance that operators will not meet again. For example, if each sequence is only half as important as the previous one, then δ=1/2. Suppose that operators harmoniously coexist with a whole stage of mutual cooperation, by getting $U_{sh}$ at each sequence. Then, the cumulative value of every sequence is $U_{sh}+U_{sh}/2+U_{sh}/4+\cdots =2U_{sh}$. To compare the payoff with the one-shot game, one might multiply future discounted reward in Equation (1) by 1−δ to normalize it as $U_{sh}$. Now we investigate two operators’ interaction in the unlicensed spectrum in an infinite number of repeated games.

#### 3.4.1. Harmonious Coexistence by Always Cooperation

Suppose LTE operator 1 and LTE operator 2 are following the policy of always LTE with LBT, e.g., always cooperation (AC) is one of the strategies. In game theory, a strategy is generally a specification of what to do in any situation that might arise. Herein, in the infinite repeated game, we use the term strategy to describe a certain policy or behavior pattern expected from an operator in response to another’s action at the *latest* stage.

A well-known example of many strategies is grim trigger, i.e., an operator cooperates as long as the other has been cooperating, and defects as soon as the other deviates. Although the grim trigger strategy is extremely harsh toward its counterpart, we can show the condition of δ that no operators ever want to deviate from AC as long as everyone has been cooperating. To show this, the utility from mutual cooperation must be greater than or equal to that from temptation to defect in an infinitely repeated game. If two operators stick to AC, the utility is $U_{sh}+\delta U_{sh}+\delta^{2}U_{sh}+\delta^{3}U_{sh}+\cdots =U_{sh}/\left(1-\delta \right)$. However, once one operator defects at the first stage, then the other operator defects according to the grim trigger strategy. The utility of the betrayer is $U_{s}+\delta U_{f}+\delta^{2}U_{f}+\delta^{3}U_{f}+\cdots =U_{s}+\delta U_{f}/\left(1-\delta \right)$. The difference between the utilities in two cases is $U_{sh}-U_{s}+\delta \left(U_{sh}-U_{f}\right)+\delta^{2}\left(U_{sh}-U_{f}\right)+\delta^{3}\left(U_{sh}-U_{f}\right)+\cdots =U_{sh}-U_{s}+\delta \left(U_{sh}-U_{f}\right)/\left(1-\delta \right)$. Thus, difference is non-negative if $U_{sh}-U_{s}+\delta \left(U_{sh}-U_{f}\right)/\left(1-\delta \right)\geq 0$, e.g., $\delta \geq \left(U_{s}-U_{sh}\right)/\left(U_{s}-U_{f}\right)$.

Hence, as long as operators care about the next period at least $\left(U_{s}-U_{sh}\right)/\left(U_{s}-U_{f}\right)$ as much as the current stage, AC can be mutually induced. Note that AC with the grim trigger strategy is the most strict and unforgiving strategy for operators in the unlicensed spectrum game. Although operators seem to adhere to AC based on the grim trigger strategy, in reality everlasting punishment is too harsh to efficiently and harmoniously utilize the unlicensed spectrum. There is a high possibility of multi-RAT networks unintentionally interfering with incumbent systems by miss detection and false alarm. We will discuss monitoring and punishment issues for a selfish network in the next subsection.

#### 3.4.2. Collusion between Two LTE Operators

So far, the payoffs are ordered by $U_{s}>U_{sh}>U_{f}>U_{w}$, and these inequalities generally hold when we observe the behavior of operators. Now we check whether the game with two operators in the unlicensed spectrum satisfies $U_{sh}>\left(U_{s}+U_{w}\right)/2$ or not. As long as the inequality holds, it prevents operators from choosing the strategy of *alternating cooperation and defection* which results in greater utility than mutual cooperation in the repeated game. Unfortunately, if we regard payoffs of operators as their channel capacity, the inequality does not always hold. For two operators, the intuition is that $U_{w}$ is simply zero but $U_{sh}$ cannot even satisfy $U_{sh}=U_{s}/2$, theoretically and experimentally. This is because sharing the unlicensed spectrum always causes medium access control (MAC) overhead and high collision probability due to CCA such as LBT schemes and CSMA/CA, unless a unified central scheduler can coordinate multi-RAT networks in every synchronous time slot. Moreover, as the number of WiFi networks increases in the co-channel of the unlicensed spectrum, LTE operators can notice that their portion of actual transmission time substantially decreases.

**Figure 4:**
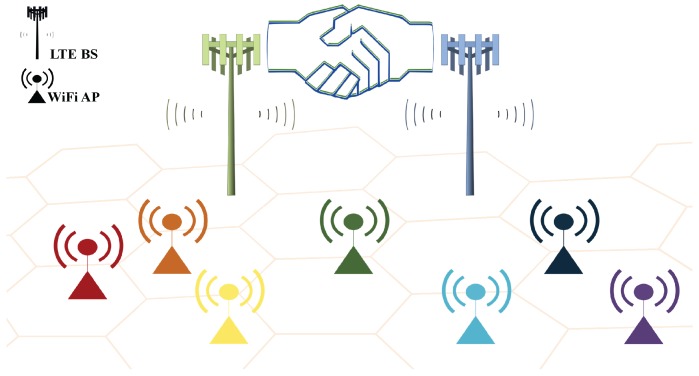
A scenario of collusion between two LTE operators under high density heterogeneous networks in the co-channel of the unlicensed spectrum.

The dense deployment of WiFi networks makes it possible for a few LTE operators to collude with each other under adverse circumstances as in Figure 4. As an example of the worst case, if two operators have $U_{sh}$ that is almost equivalent to $U_{f}$ due to highly busy channels, then $U_{sh}\ll \;\left(U_{s}+\;U_{w}\right)/2$ is enough to steer them towards disharmonious and corrupt behavior, e.g., in a country without LBT requirements, LTE operators are highly susceptible to collusion to exploit scarce spectrum resources by deceiving other networks into believing that channels are extremely busy. In a similar way to the always cooperation strategy, when $U_{sh}<\left(U_{s}+U_{w}\right)/2$ holds, the condition of δ for collusion between two LTE operators can also be derived by using the grim trigger strategy.

#### 3.4.3. Tit for Tat between Two LTE Operators

When LTE operators in the unlicensed spectrum regard $U_{w}$ not as zero, but as a negative value because of delay-sensitive applications and users, $U_{sh}\;>\;\left(U_{s}+\;U_{w}\right)/2$ induces them to use many different strategies in an infinite or unknown length game. Perhaps LTE operators want to know what kind of strategies will produce the highest possible utility.

According to Proposition 1 in [27], if δ is sufficiently large, no best decision rule exists independently of the strategy being used by the other player in the iterated prisoners’ dilemma. However, when we apply this statement to the operator’ dilemma, it does not necessarily mean that this dilemma cannot be resolved or that operators are always tempted to cheat in order to maximize their utility. Although there is no one best strategy, *tit for tat* (TFT) may be an effective strategy for two LTE operators in the co-channel of the unlicensed spectrum. TFT is a decision rule of cooperating at the first stage, and then always repeating what the other operator has done at the previous stage. TFT was first introduced as a strategy by Anatol Rapoport in Robert Axelrod’s two tournaments [27]. Interestingly, in the first and second rounds, TFT was the most successful strategy by getting a higher cumulative payoff in the entire round robin tournament. Although professional game theorists and researchers in many different fields were invited to submit their favorite strategies for computer tournaments, the winner was the simplest strategy: the TFT strategy. The reasons for TFT’s victory in these tournaments were analyzed as follows: (1) cooperation as long as the other player cooperates by avoiding unnecessary conflict; (2) retaliation for unexpected defection by the other; (3) forgiveness by cooperating with the other in spite of historical background; (4) clarity for the other to learn the TFT’s behavior pattern quickly. More information on tournament results can be found in [27].

**Figure 5:**
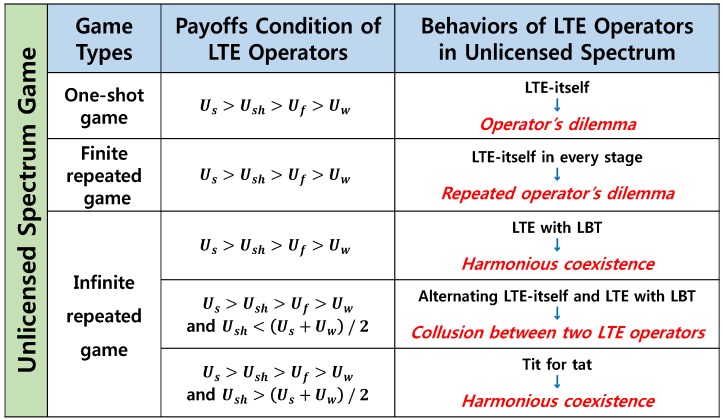
The results of the unlicensed spectrum game: LTE operators have strategic plans for the unlicensed spectrum.

When $U_{s}>U_{sh}>U_{f}>U_{w}$ and $U_{sh}\;>\;\left(U_{s}+\;U_{w}\right)/2$ are assumed, TFT is stable and credible for two LTE operators if and only if δ is greater than $\left(U_{s}-U_{sh}\right)/\left(U_{sh}-U_{w}\right)$ and $\left(U_{s}-U_{sh}\right)/\left(U_{s}-U_{f}\right)$, e.g., TFT is called a Nash equilibrium (NE). However, TFT is generally not a subgame perfect Nash equilibrium (SPNE) that represents a NE of every subgame of the original game. One can refer to [25] for a detailed discussions on *contrite tit for tat* known as a SPNE variant of TFT as well as for the basic concept of game theory. Finally, we summarize the results in Figure 5.

#### 3.4.4. Performance Analysis for Multi-RAT Systems

Based on a potential collusion scenario and a harmonious coexistence scenario, we now investigate the performance of both LTE and WiFi networks to point out the importance of mandatory LBT. In performance analysis, we consider payoffs as the average channel utilization in the unlicensed spectrum [19]. LTE operators are assumed to utilize the 3GPP category 4 LBT scheme to achieve fair coexistence with WiFi networks because its sharing mechanism operates as WiFi-like as possible among existing LBT schemes such as ETSI, ECCA, and ICCA [17]. Both LTE and WiFi have two different traffic models—File Transfer Protocol (FTP) traffic and Voice over Internet Protocol (VoIP) traffic—which indicate low load and high load, respectively [20]. We also evaluate the average channel utilization of both LTE and WiFi networks as the number of LTE operators increases.

**Figure 6:**
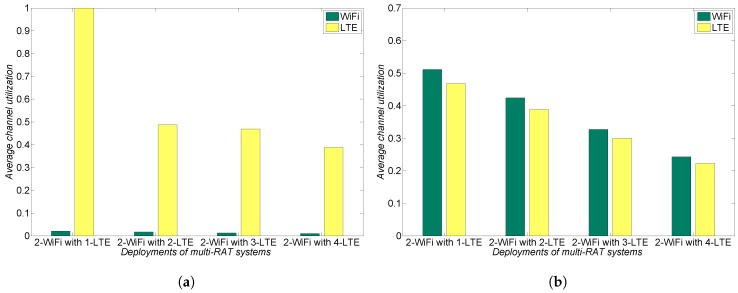
Average channel utilization with low load. (**a**) potential collusion between LTE operators without mandatory LBT; and (**b**) LTE operators with mandatory LBT towards harmonious coexistence.

Figure 6 represents the average channel utilization when LTE and WiFi networks have low load. If LBT is not mandatory, LTE operators possibly collude with each other and then deceive two WiFi networks into thinking that the unlicensed spectrum is always busy. As shown in Figure 6a, as the number of LTE operators increases, they alternatively exploit the unlicensed spectrum in their own selfish manner, e.g., a round robin between LTE operators. In this case, WiFi networks cannot use the unlicensed spectrum at all. In Figure 6b, once LBT is mandated to induce reciprocal behavior from LTE operators, the performance of WiFi networks significantly improves as much as that of LTE operators. Therefore, a mandatory LBT enables both LTE operators and the WiFi network to achieve harmonious coexistence in the unlicensed spectrum.

**Figure 7:**
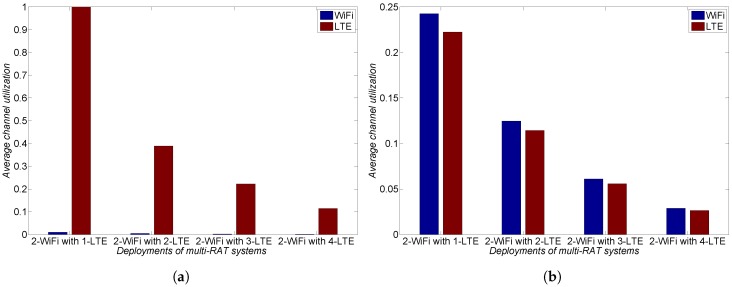
Average channel utilization with high load. (**a**) potential collusion between LTE operators without mandatory LBT; and (**b**) LTE operators with mandatory LBT towards harmonious coexistence.

In a similar way, Figure 7 presents average channel utilization with high load. As the number of LTE operators increases, although LTE operators exploit the unlicensed spectrum based on their own cooperation, fair coexistence with WiFi networks hardly happens without a mandatory LBT in Figure 7a. As shown in Figure 7b, a mandatory LBT of LTE operators is essential for mutual benefits towards harmonious coexistence between LTE operators and WiFi networks.

## 4. Discussion, Future Topics, and Conclusions

### 4.1. Discussion

So far, we have examined how two LTE operators carry out strategic plans for the unlicensed spectrum under different assumptions of their payoffs. Since the spectrum is scarce and valuable, the unlicensed spectrum should be preserved as common resources so that any wireless networks can operate their own service. After Qualcomm proposed LTE-U to deliver LTE with small cells using the unlicensed 5-GHz spectrum, many companies in the wireless industry and 3GPP have continued to work towards the standardization of LTE-LAA. To coexist harmoniously with other wireless networks, the LBT mechanism is included in LTE Release 13.

Apart from technical standards, we have verified that LTE operators might as well strategically exploit the unlicensed spectrum under dense heterogeneous networks in countries without specific requirements for the usage of LBT. Based on the operator’s dilemma game, we highly recommend that telecommunications regulatory bodies mandate the usage of LBT in the 5 GHz band at national level. Otherwise, different LTE operators have an incentive to advance their network strategies to maximize utility in the unlicensed spectrum. Moreover, if possible, it would be better to encourage every LTE operator to unify the LBT mechanism. Different LBT configurations may cause a high collision rate and severe overhead, and consequently the overall spectral efficiency will decrease.

In addition to regulatory requirements of the LBT mechanism, it is necessary to detect and monitor malicious wireless users who violate power limits or produce jamming signals. During a certain period of time, a monitoring system must look at the behavior patterns in the unlicensed spectrum and inflict punishment on selfish wireless networks in order to achieve harmonious coexistence. Data from the monitoring system could help regulatory bodies to propose a new frequency allocation and channel assignment plan in the unlicensed spectrum. For example, some countries are moving toward allowing an unmanned aerial vehicle (UAV), typically known as a drone, to use the unlicensed spectrum within the 5 GHz band for remote-control communications. Under severe interference conditions, drones and other wireless systems cannot expect reliable communications. For this reason, recently, Korea allowed drones to use a dedicated spectrum 5030–5091 MHz (61 MHz bandwidth) for commercial purposes only, and planned for an additional 160 MHz bandwidth within the 5 GHz band.

### 4.2. Future Topics

LBT-Advanced: According to average user throughput results in [14], when a single LTE operator uses the coexistence mechanism in the unlicensed spectrum, different LBT schemes affect the collision probability with WiFi networks. Consequently, resource allocation between WiFi and LTE networks in the co-channel highly depends on CCA time, CCA period, and channel occupancy time based on a specific LBT feature. To better coexist with WiFi networks, an LTE operator may propose an advanced and unified version of LBT to make WiFi networks consider the LTE system as WiFi itself. Hence, multiple LTE operators with LBT-Advanced (LBT-A) enable all users from multi-RAT systems to benefit from the reduced collision probability and improved spectral efficiency.

Uplink LBT: LTE in the unlicensed spectrum currently focuses on downlink transmission because data traffic of downlink is generally much heavier than that of uplink. When the unlicensed spectrum is used for uplink transmission, user equipment (UE) will carry out an LBT procedure before transmitting over target uplink channels. In this situation, multi-path fading, shadowing, and the hidden node problem may cause serious degradation of miss detection and false alarm performance. In addition to reliable LBT, energy-efficient LBT methods are necessary especially for power-constrained UEs. To this end, the cooperative LTE mechanism is significantly beneficial to the mitigation of these issues. Since many cooperative sensing schemes have shown reliable and energy-efficient performance in cognitive radio, LTE compliant cooperation and the data fusion process are interesting research topics for uplink LBT (UL LBT) of enhanced LAA (Release 14, 15, and further versions) to protect incumbent systems.

Modeling complicated behavior by game theory: The operator’ dilemma is a typical non-cooperative game where operators only care about their own benefit and choose the best strategy to maximize their payoff function. One can extend the operator’s dilemma to describe such a scenario where strategies such as LTE-itself and LTE with LBT are non-deterministic, e.g., a solution concept is the mixed strategy Nash equilibrium. In addition to the non-cooperative game, one can design a cooperative LBT game to analyze the strategic interaction between base stations for downlink LBT (DL LBT) as well as users for UL LBT. Hence, it is necessary for operators in the game to have an agreement on how to utilize and distribute unlicensed spectrum resources based on their own contributions. This bargaining game is useful to verify the enforceable requirements of CCA time, CCA threshold, CCA consumption power, and expected occupancy time to maximize their utilities respectively.

### 4.3. Conclusions

In this article, we have verified the payoffs of multi-RAT systems in the unlicensed spectrum and introduced the operator’s dilemma together with WiFi deployment. The behaviors of operators were investigated from both short- and long-term points of view. We concluded that mandatory usage of LTE with LBT is highly recommended to achieve harmonious coexistence in the unlicensed spectrum because, in countries without LBT requirements, one LTE operator possibly colludes with the other LTE operator to extremely exploit scarce spectrum resources by deceiving other networks into thinking that channels are busy.
